# Supplementary material for: Automated Machine Learning Pipeline: Large Language Models-Assisted Automated Data set Generation for Training Machine-Learned Interatomic Potentials
Source: J Chem Theory Comput. 2025 Dec 26;22(1):305–17. doi: 10.1021/acs.jctc.5c01610 (PMC12805562; doi:10.1021/acs.jctc.5c01610)
Supplement: Supplementary file 1 [file ct5c01610_si_001.pdf]

Supporting Information:

Automated Machine Learning Pipeline: Large Language Models-Assisted Automated Dataset Generation for Training Machine-Learned Interatomic Potentials.

Adam Lahouari[1,*], Jutta Rogal[1,2], and Mark E. Tuckerman[1,3,4,5,6]

[1]NYU, Department of Chemistry, New York, NY 10003, USA

[2]Initiative for Computational Catalysis, Flatiron Institute, NY 10010, USA

[3]NYU, Department of Physics, New York, NY 10003, USA

[4]Courant Institute of Mathematical Sciences, NYU, NY 10012, USA

[5]NYU-ECNU Center for Computational Chemistry, Shanghai 200062, China

[6]Simons Center for Computational Physical Chemistry, NYU, NY 10003, USA

[*]Correspondence: al9500@nyu.edu

Key Findings from AMLP Literature Analysis

Table S1 summarizes the major insights identified by the multi-agent literature analysis, with direct links to the retrieved publications and expert assessments.

Table S1: Key findings from AMLP multi-agent literature analysis for acridine polymorph research.

Finding Category	Key Insight	Source & Evidence
Acridine Literature	Limited direct studies on acridine polymorphs	Experimental & theoretical reports: no specific acridine polymorph literature found
Crystal Structure Prediction	AlphaCrystal-II deep learning approach	`arXiv:2404.04810v1` – distance matrix-based crystal prediction
Experimental Techniques	PXRD for crystal characterization	`arXiv:2401.03862v3` – end-to-end crystal structure prediction from PXRD
Novel Analysis Methods	Inverse EXAFS Analysis (IEA)	`arXiv:2409.09693v1` – comparative study with Demeter software
Materials Discovery	Autonomous ML-driven discovery	`arXiv:2508.02956v1` – multi-agent physics-aware reasoning
Similar Systems	Aromatic heterocycle precedents	Theoretical report: anthracene, phenanthrene, quinoline as model systems
Computational Gaps	DFT + ML integration needed	All expert reports: lack of systematic acridine polymorph studies
Methodology Limitations	Self-interaction error in DFT	Gaussian expert: `arXiv:2412.18350v1` – exchange-correlation functionals
Software Capabilities	VASP for solid-state calculations	VASP expert: `arXiv:2508.07035v1` – VASPilot automation platform
Force Field Development	Neural network potential comparison	VASP expert: `arXiv:2304.10820v1` – Matlantis vs VASP comparison

Research Opportunity Assessment

Based on the literature analysis, AMLP identified several research opportunities:

Table S2: Research gaps and opportunities identified by AMLP for acridine ML potential development.

Gap Type	Specific Opportunity	Recommended Approach
Experimental Data	Acridine polymorph characterization	Systematic PXRD and thermal analysis studies
Computational Benchmark	DFT method validation for acridine	Compare PBE, PBE-D3(BJ), hybrid functionals
ML Architecture	Neural network potential training	Combine VASP DFT data with modern ML frameworks
Phase Transition	Thermodynamic modeling	Free energy calculations using enhanced sampling
Software Integration	Automated workflow development	Extend VASPilot-type approaches to polymorph screening
Validation Framework	Experimental-computational synergy	Cross-validate ML predictions with crystal growth experiments

Table S3: Actual AMLP DFT-parameter recommendations for acridine crystalline systems.

Software	Parameter	Recommended Value & Provenance
VASP	ENCUT	500 eV (molecular systems literature)
	XC functional	PBE (standard for organic crystals)
	Method focus	Periodic DFT with dispersion corrections
Gaussian	Method	B3LYP (organic chemistry standard)
	Basis set	6-31G(d,p) (balanced accuracy/cost)
	Application	Molecular property benchmarking
CP2K	XC functional	PBE (consistent with VASP)
	Basis set	DZVP-MOLOPT-SR-GTH
	Method	Mixed Gaussian/plane-wave (GPW)
General	Dispersion	D3(BJ) corrections recommended
	Convergence	10^{-6} eV electronic, 10^{-3} eV Å^{-1} forces

Computational Methods

Density Functional Theory (DFT) Calculations

All DFT calculations were performed using the Vienna Ab initio Simulation Package (VASP) with the Projector Augmented Wave (PAW) method.[18–21] In this study on acridine, we decided to use VASP with the following parameters:

Cell Optimization Parameters

Table S4: VASP parameters for cell optimization

Parameter	Value	Description
SYSTEM	acridine_cell	System identifier
ALGO	Normal	Electronic minimization algorithm
EDIFF	1×10^{-6} eV	Electronic convergence criterion
ENCUT	850.0 eV	Plane-wave cutoff energy
GGA	PE	Exchange-correlation functional (PBE)
IBRION	2	Ionic relaxation algorithm (conjugate gradient)
ISIF	3	Stress tensor calculation (cell shape and volume optimization)
ISMEAR	-1	Smearing method (Fermi smearing)
IVDW	13	van der Waals correction method
KPOINTS	7×7×7	K-point mesh (Gamma-centered, automatic)
LCHARG	.TRUE.	Write charge density
LREAL	Auto	Real-space projection
LVDW	.TRUE.	Enable van der Waals corrections
LWAVE	.TRUE.	Write wavefunctions
NELM	100	Maximum electronic self-consistency steps
NSW	150	Maximum ionic relaxation steps
PREC	High	Precision level
SIGMA	0.05 eV	Smearing parameter
VDW_R0	12.0 Å	van der Waals cutoff radius

Ab Initio Molecular Dynamics (AIMD) Parameters

For the AIMD, the following parameters were used for different temperature:

Table S5: Molecular dynamics control parameters for AIMD simulation at 300 K

Parameter	Value	Description
IBRION	0	Molecular dynamics mode
NSW	10,000	Number of MD steps
POTIM	1.0 fs	Time step
TEBEG	300.0 K	Initial temperature
TEEND	300.0 K	Final temperature
ISYM	0	Symmetry disabled for MD
MDALGO	3	Langevin thermostat
ISIF	2	Stress tensor calculation (ionic positions only)

Langevin Thermostat Parameters:

- **LANGEVIN_GAMMA**: 10.0 10.0 10.0 (friction coefficients for N, C, H atoms)
- **LANGEVIN_GAMMA_L**: 1.0 (lattice friction coefficient)
- Species-specific friction coefficients: N: $\gamma = 10.0\,\text{THz}$, C: $\gamma = 10.0\,\text{THz}$, H: $\gamma = 10.0\,\text{THz}$

Table S6: Electronic structure and algorithm parameters for AIMD

Parameter	Value	Description
ENCUT	850.0 eV	Plane-wave cutoff energy
EDIFF	1×10^{-6} eV	Electronic convergence criterion
NELM	100	Maximum electronic steps
ISMEAR	-1	Fermi smearing
SIGMA	0.05 eV	Smearing parameter
GGA	PE	PBE exchange-correlation functional
LVDW	.TRUE.	Enable van der Waals corrections
IVDW	13	van der Waals method
VDW_R0	8.0 Å	van der Waals cutoff radius
PREC	High	Precision level
ALGO	Fast	Electronic minimization algorithm
LREAL	Auto	Real-space projection
LWAVE	.FALSE.	Do not write wavefunctions
LCHARG	.FALSE.	Do not write charge density
NBLOCK	1	Output frequency
KBLOCK	10	k-point output frequency
LCHIMAG	.FALSE.	Magnetic moments output

Machine Learning Model Training

Data Preparation

For the training dataset preparation:

- **Force cutoff**: 10.0 eV Å^{-1} (configurations with forces exceeding this threshold were excluded from training)
- **Atomic species**: H ($Z = 1$), C ($Z = 6$), N ($Z = 7$)
- **Data split**: 85% training, 15% validation

MACE Model Configuration

The Machine learning Atomic Cluster Expansion (MACE) model was trained using a foundation model approach with the following configuration:

Table S7: MACE model architecture and training parameters

Parameter	Value	Description
**Foundation Model Settings**		
foundation_model	mace-mpa-0-medium.model	Base pre-trained model
multiheads_finetuning	False	Transfer learning approach
**Model Architecture**		
num_channels	128	Number of hidden channels
max_L	2	Maximum angular momentum
num_interactions	2	Number of interaction layers
correlation	3	Correlation order
max_ell	3	Maximum spherical harmonic degree
r_max	6.0 Å	Cutoff radius
**Training Parameters**		
device	cuda	Training device
batch_size	12	Training batch size
max_num_epochs	350	Maximum training epochs
default_dtype	float64	Numerical precision
**Optimization Settings**		
swa	True	Stochastic Weight Averaging
start_swa	250	SWA start epoch
swa_lr	0.0001	SWA learning rate
swa_forces_weight	10	SWA forces weight
swa_energy_weight	1	SWA energy weight
ema	True	Exponential Moving Average
ema_decay	0.99	EMA decay rate
amsgrad	True	AMSGrad optimizer variant
**Loss Function and Scaling**		
loss	ef	Energy and forces loss
scaling	rms_forces_scaling	Force scaling method
error_table	PerAtomMAE	Error metric

Atomic Reference Energies

Table S8: Atomic reference energies (E0s) used in MACE training

Element	Energy (eV)
H	-0.27737421
C	-0.77091629
N	-0.83778712

Ensemble Training

To ensure robustness and estimate model uncertainty, three independent models were trained using different random seeds with identical hyperparameters:

- **Seeds**: 49, 67, 127
- **Training epochs**: 300 epochs each
- **Committee approach**: All models trained with identical hyperparameters to form an ensemble for improved prediction accuracy and uncertainty quantification
- **Total number of parameters**: 752,174

Energy Conservation

The corresponding cumulative energies are summarized in Figure S1. For these simulations, all unit cells were replicated, reaching approximately 25 Å in each direction and resulting in system sizes ranging from 1,104 to 2,944 atoms, depending on the polymorph. To achieve this, a cell-replication tool was implemented within the AMLP-A module, enabling users to easily apply scaling factors along specified directions.

Across all 24 simulations (3 committees × 8 polymorphs), the total energies are well conserved with an average energy drift consistently on the order of 10^{-4}. This performance confirms that the trained potentials are robust for energy conservation.

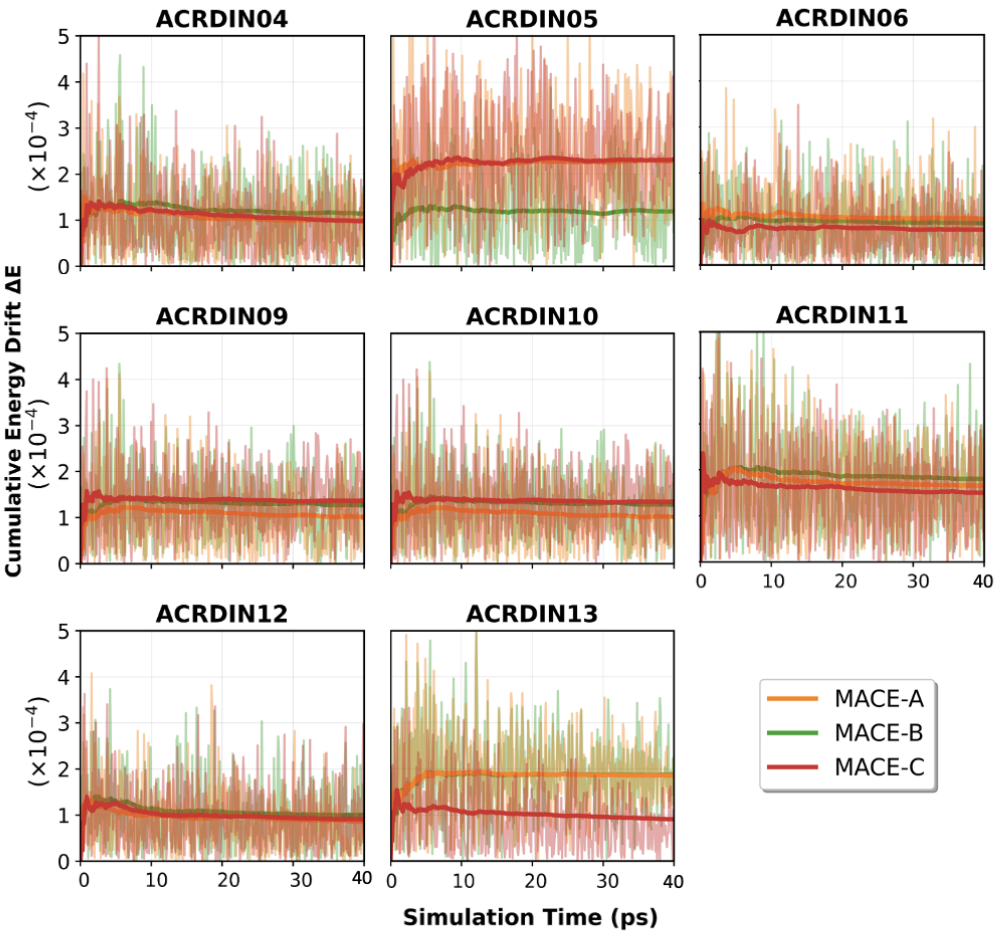

Figure S1: Cumulative energy conservation from NVE simulations of 39 ps following 1 ps of equilibration across all eight acridine polymorphs. Each panel corresponds to one polymorph, with results shown for three independently trained MLIPs: MACE-A (orange), MACE-B (green), and MACE-C (red). Solid lines represent the cumulative energy conservation, while shaded fluctuations reflect instantaneous deviations. All simulations show energy conservation on the order of 10^{-4}.

Interestingly, minor variations across polymorphs are observed. For example, ACRDIN04, ACRDIN06, ACRDIN09, ACRDIN10, ACRDIN11, and ACRDIN12 consistently exhibited very good energy conservation across all committees. By contrast, MACE-B produced the lowest variation for ACRDIN05, while MACE-C was slightly better for ACRDIN13. These subtle differences suggest that structural features of individual polymorphs may influence energy conservation.

Supplementary Figures

Radial Distribution Functions: C–N pairs

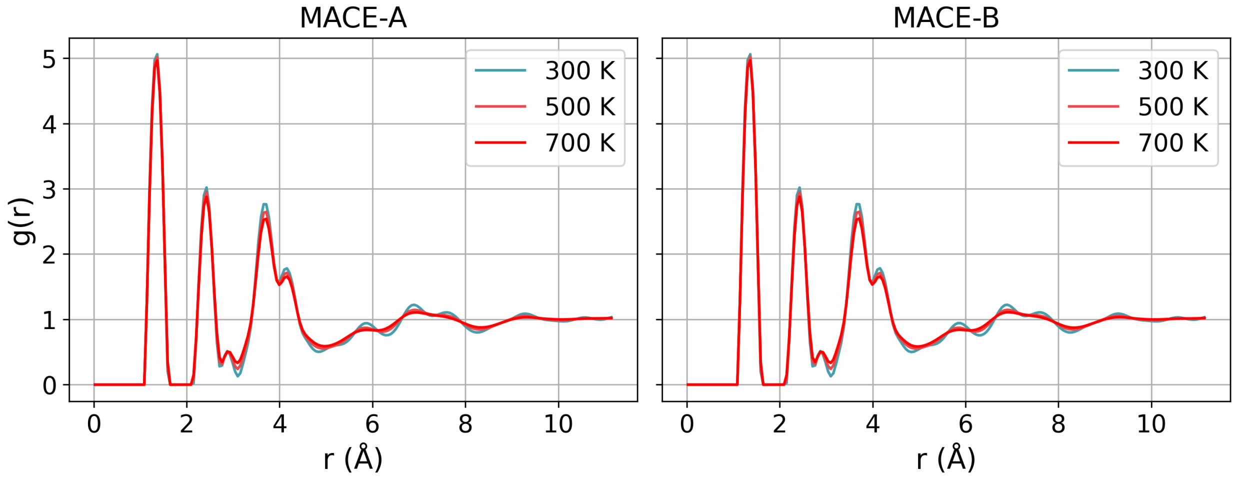

Figure S2: C–N pair radial distribution function $g_{\mathrm{C–N}}(r)$ for ACRDIN04 acridine polymorph.

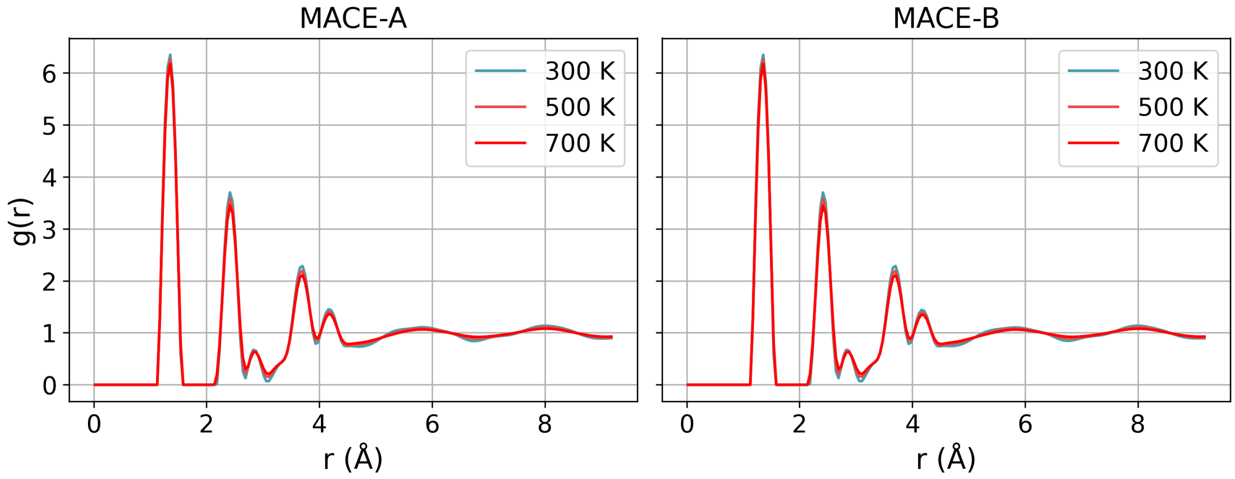

Figure S3: C–N pair radial distribution function $g_{\mathrm{C–N}}(r)$ for ACRDIN05 acridine polymorph.

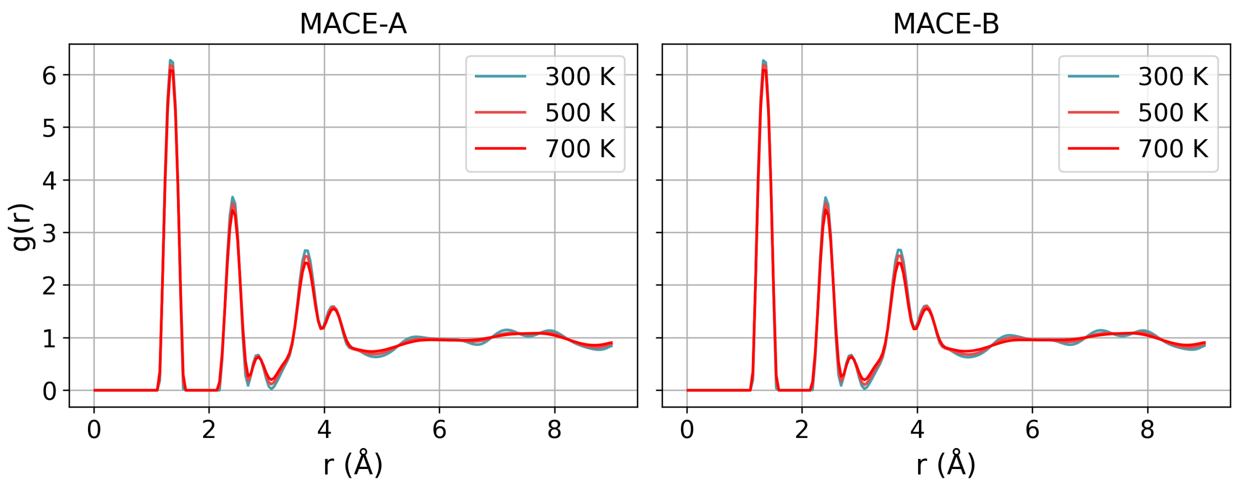

Figure S4: C–N pair radial distribution function $g_{\text{C–N}}(r)$ for ACRDIN06 acridine polymorph.

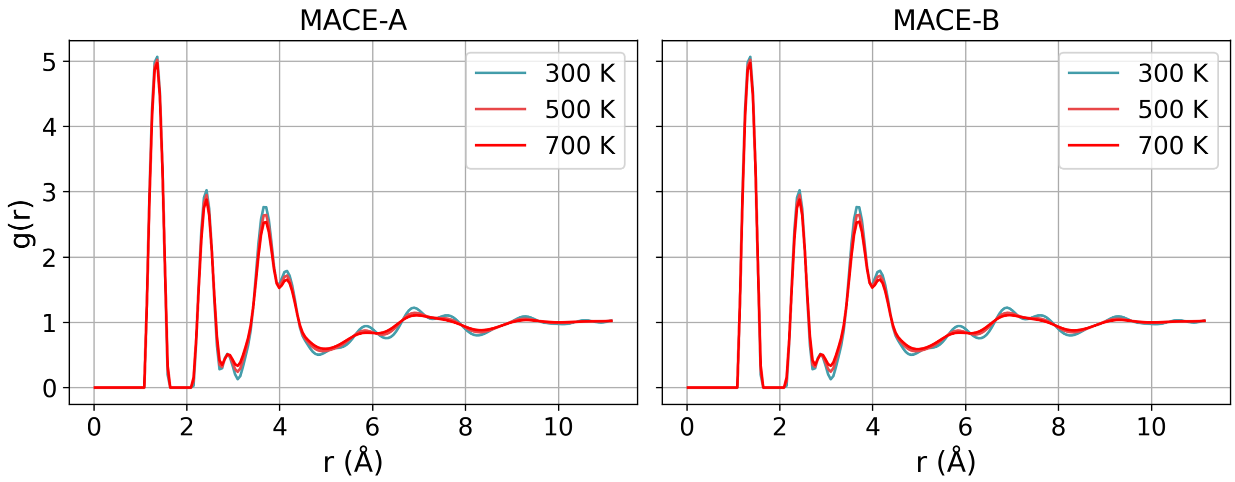

Figure S5: C–N pair radial distribution function $g_{\text{C–N}}(r)$ for ACRDIN11 acridine polymorph.

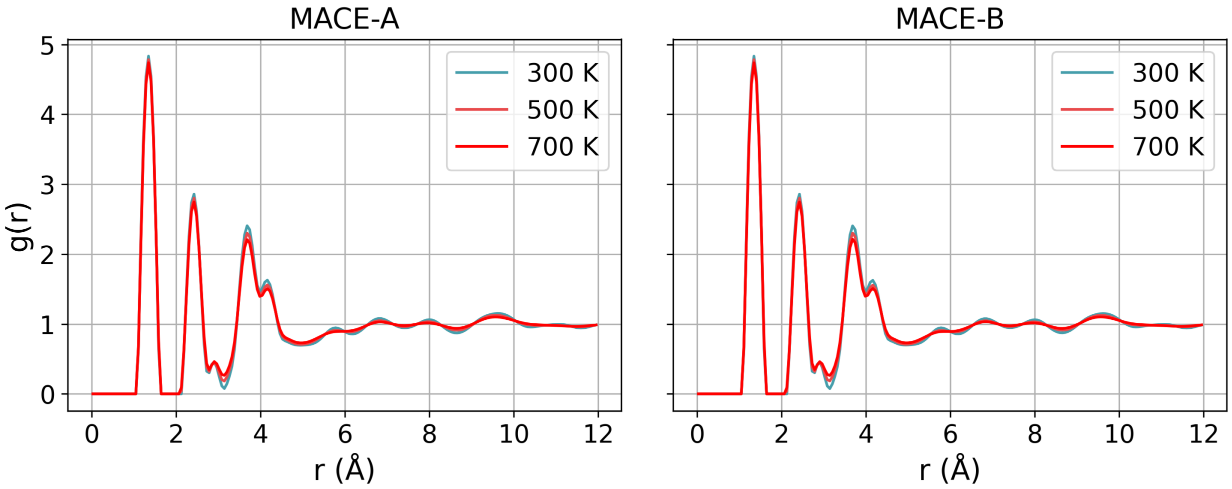

Figure S6: C–N pair radial distribution function $g_{\text{C–N}}(r)$ for ACRDIN13 acridine polymorph.

Radial Distribution Functions: N–N pairs

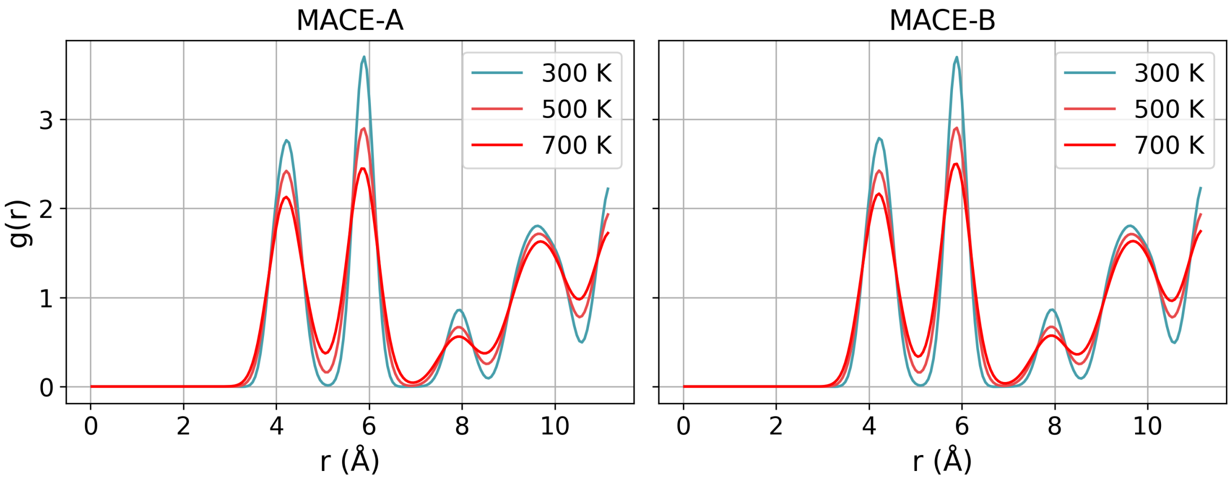

Figure S7: N–N pair radial distribution function $g_{\text{N–N}}(r)$ for ACRDIN04 acridine polymorph.

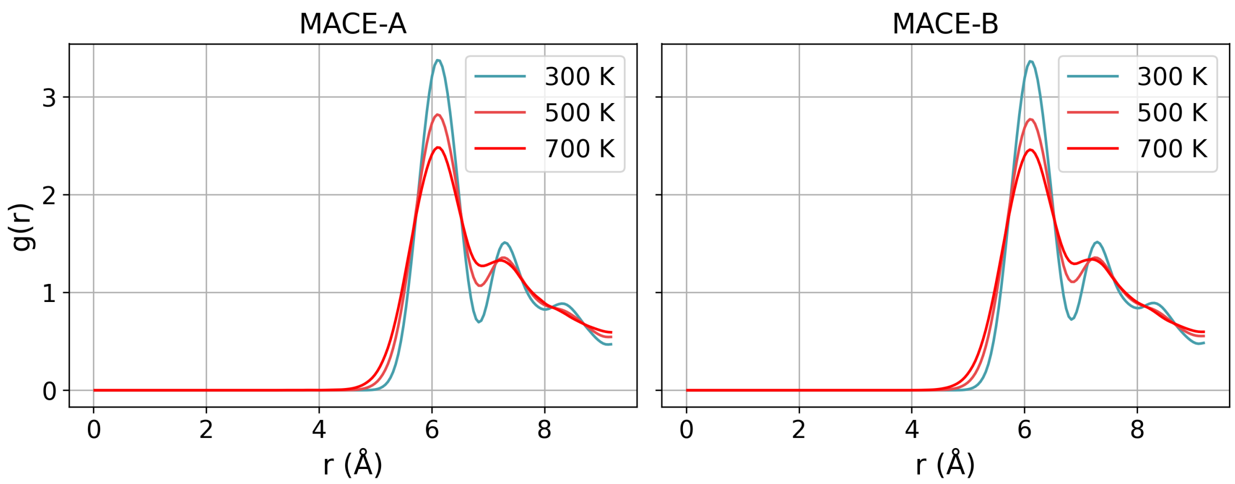

Figure S8: N–N pair radial distribution function $g_{\text{N–N}}(r)$ for ACRDIN05 acridine polymorph.

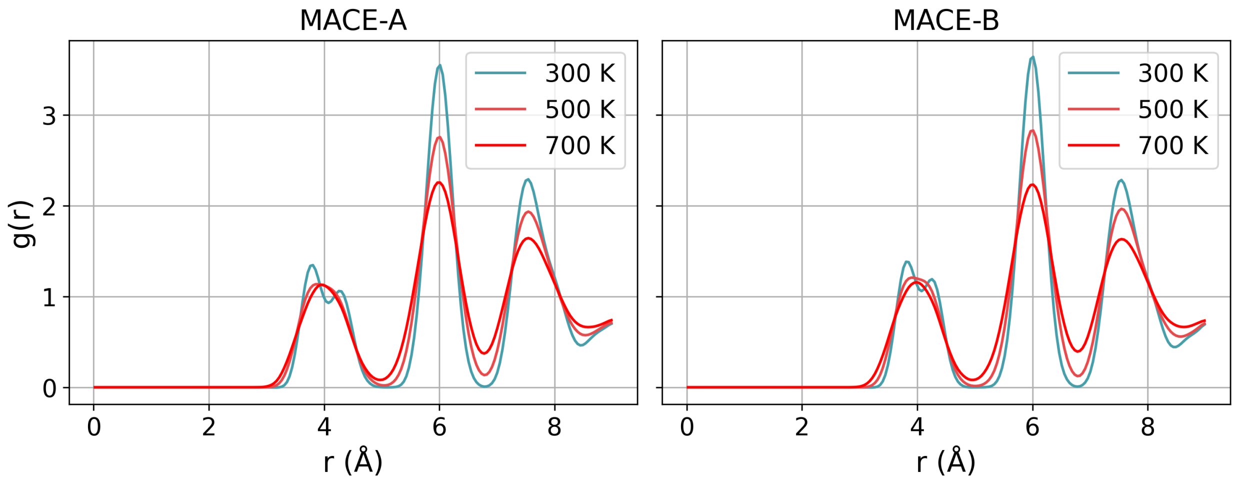

Figure S9: N–N pair radial distribution function $g_{\text{N–N}}(r)$ for ACRDIN06 acridine polymorph.

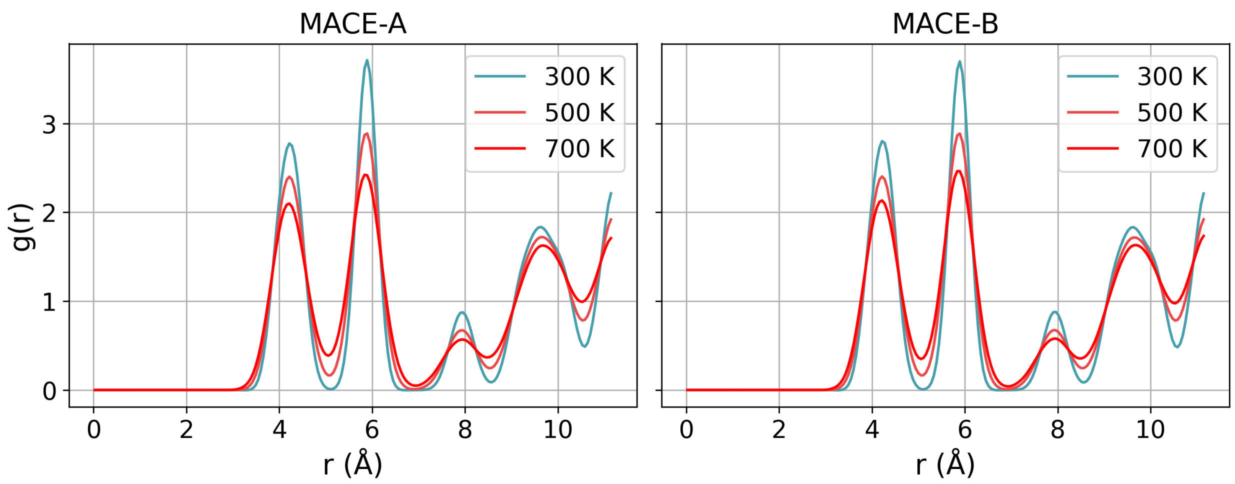

Figure S10: N–N pair radial distribution function $g_{\mathrm{N–N}}(r)$ for ACRDIN11 acridine polymorph.

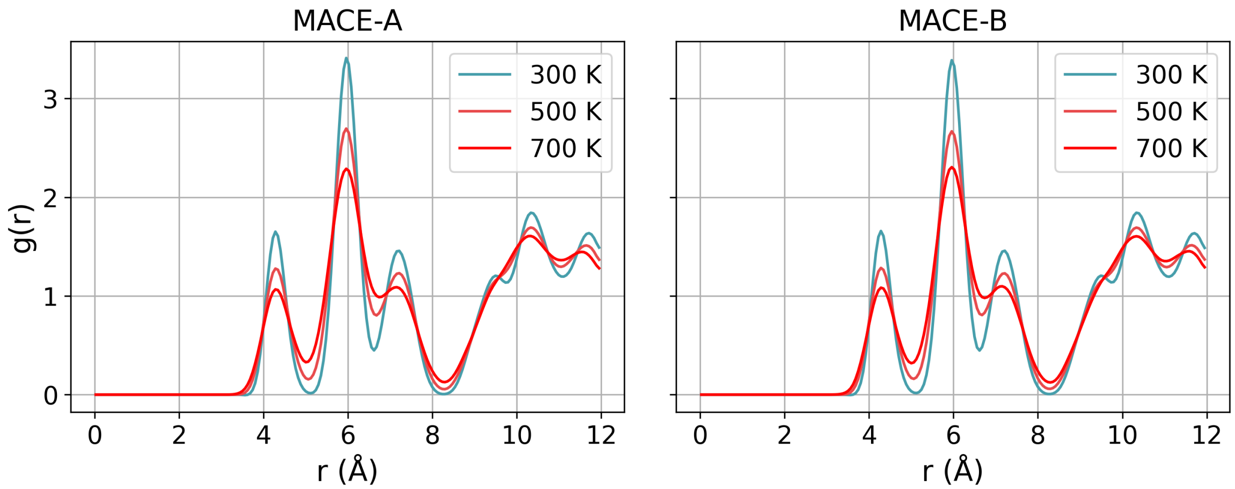

Figure S11: N–N pair radial distribution function $g_{\mathrm{N–N}}(r)$ for ACRDIN13 acridine polymorph.

Out-of-Sample Validation: ACRIDIN VIII

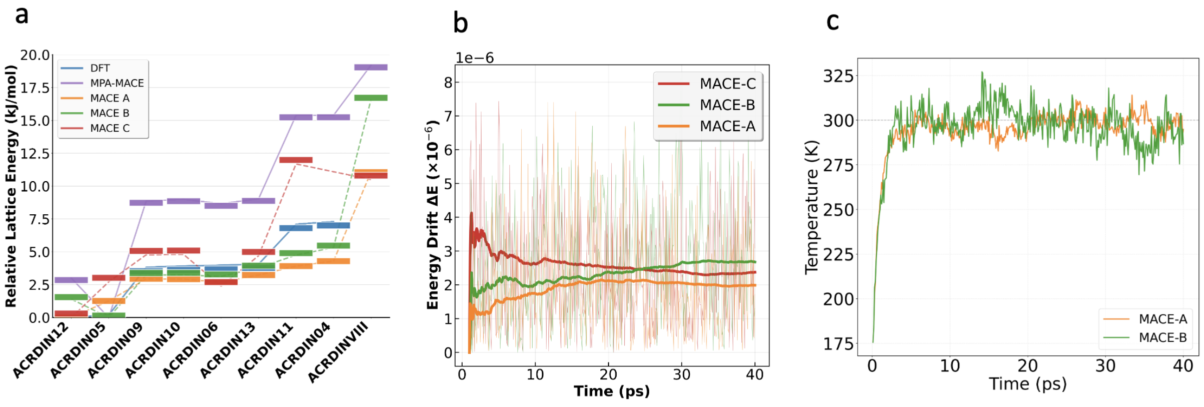

Figure S12: Performance evaluation of MACE committee models on ACRIDINVIII, a polymorph excluded from the training set. (a) Relative lattice energies of acridine polymorphs after geometry optimization, comparing DFT reference calculations with MPA-MACE and individual committee models (MACE-A, MACE-B, MACE-C). ACRIDINVIII (Form VIII) was not included in the training data. (b) Energy drift assessment during 40 ps NVE molecular dynamics simulations at 300 K using the three MACE committee models, demonstrating energy conservation with MACE-C showing the lowest drift (ΔE 2.0×10^{-6} eV/atom). (c) Temperature stability during 40 ps NVT simulations at 300 K for MACE-A and MACE-B committees, showing equilibration around the target temperature with minimal fluctuations.
